# Gender differences among psychiatric patients attended in a emergency department

**DOI:** 10.1192/j.eurpsy.2021.1600

**Published:** 2021-08-13

**Authors:** M. Recio-Barbero, A. Santorcuato, A. Bacigalupe, M. López-Atanes, S. Fuertes-Soriano, J. Cabezas-Garduño, J.P. González-Briceño, M. Sáenz-Herrero

**Affiliations:** 1 Psychiatry, Biocruces Bizkaia Health Research Institute- Cruces University Hospital, Barakaldo, Spain; 2 Emergency Department, Cruces University Hospital, Barakaldo, Spain; 3 Sociology Department, University of the Basque Country, Leioa, Spain; 4 Psychiatry Department, Cruces University Hospital, Barakaldo, Spain

**Keywords:** Gender, emergency psychiatry, medication, Gender bias

## Abstract

**Introduction:**

Mental health disorders are considered a priority in health policies around the world. It is estimated that more than 900 million people worldwide have a mental disorder, in which stress-related disorders account for a high number of emergency department visits. The scientific literature has pointed out the importance of considering how gender and sex differences influence the clinical outcomes of people with mental illness, playing an important role in the clinical management of these patients.

**Objectives:**

The aim of this report is to investigate the presence of gender differences in the care of psychiatric patients attending the emergency department (ED), taking into account the clinical characteristics, reasons for consultation and practices.

**Methods:**

The study considered all episodes of patients who visited the ED during 2017 and who were assessed by the psychiatric department. Statistical analyses were performed using IBM SPSS Statistic software.

**Results:**

During the 12 months period, a total of 3180 episodes were evaluated by the psychiatric department in the ED. Of them, 1723 were female (54,2%) and 1457 male. Regarding clinical data, there were found statistically significant differences with respect to the pharmacological prescription in the ED, specifically in the prescription of benzodiazepines, psychiatric diagnoses after discharge and the indication of hospital admission between women and men.

**Conclusions:**

This study emphasizes the importance of considering the existence of gender differences in both the clinical presentation as well as in the care of psychiatric patients attending the ED. The analysis of these variables would help to improve the health care of psychiatric patients.

